# 1462. Analysis of Clindamycin and Vancomycin Use and Reported Beta Lactam Allergy in Procedural Antimicrobial Prophylaxis

**DOI:** 10.1093/ofid/ofad500.1299

**Published:** 2023-11-27

**Authors:** Lisa A Veach, Amanda Bushman, Geoff Wall, Adela Hadziric, Kayla Olstinke, Sydney Blackmer

**Affiliations:** UnityPoint Health, Des Moines, Iowa; UnityPoint Health Des Moines, Urbandale, Iowa; Drake University and UnityPoint Health Des Moines, Des moines, Iowa; Unity Point Health, Urbandale, Iowa; Drake University and UnityPoint Health Des Moines, Des moines, Iowa; Drake University and UnityPoint Health Des Moines, Des moines, Iowa

## Abstract

**Background:**

Patients with a penicillin (pcn) allergy are less likely to receive cefazolin (cef) and more likely to receive vancomycin (vanco) or clindamycin (clinda) for procedures. Literature reveals a 51% increased risk of surgical site infection in patients with a reported pcn allergy attributed, in multivariable analysis, to use of second line antimicrobial agents.

**Methods:**

We conducted a retrospective review of intravenous vanco and clinda administration in procedural areas during a 6 -month period in 2022.

The aim of our study was to assess the appropriateness of the procedural prophylaxis antibiotic given based on the allergy information available in the medical record.

Documented allergy responses were classified as: severe, cutaneous and other. Multiple documented reactions were classified using the most severe reaction type.

**Results:**

Seven hundred and eighty-eight patient encounters were reviewed: 434 encounters receiving vanco and 354 encounters receiving clinda.

Of the encounters receiving vanco, 237 (55%) had documentation of beta lactam allergy (pcn allergy (212), cef allergy (64), both (39)). Within the pcn allergy group, reaction was cutaneous only in 47% (100). In 79 encounters previous receipt of a cephalosporin was documented in the EHR.

Of the encounters receiving clinda, 330 (93%) had documentation of a beta lactam allergy (pcn allergy (277), cef allergy (98), both (45)). Within the pcn allergy group, reaction was cutaneous only in 60% (167). In 109 encounters previous receipt of a cephalosporin was documented in the EHR. (Figures 1 and 2)

In both groups nursing placed the majority of the vanco (69%) and clinda (70%) orders.

**Figure:**
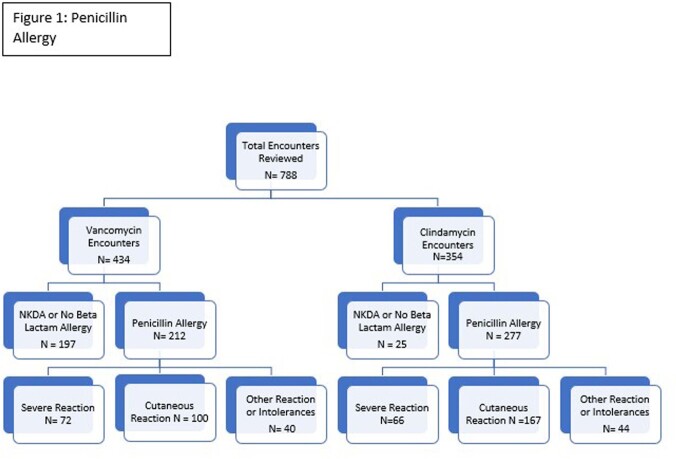

**Figure:**
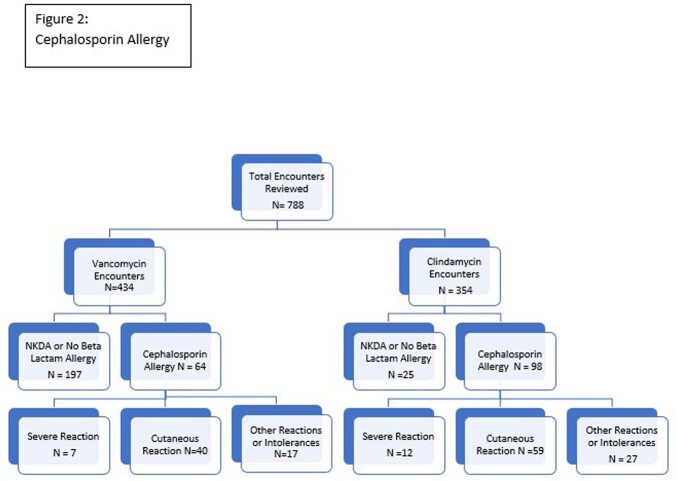

**Conclusion:**

At least 55% (N=267) of pcn allergic patients could have received the optimal agent (cefazolin) for procedural prophylaxis as they had only a cutaneous reaction. EHR review for prior cephalosporin administration in non-cutaneous reaction further increases that number.

In recognition of the role of nursing, our health system has developed guidance documents to aid pre-op nursing staff in selecting the appropriate surgical prophylaxis antimicrobial in patients reporting pcn allergy. This document is readily available and shared with key stakeholders. Process improvement opportunities are ongoing.

**Disclosures:**

**All Authors**: No reported disclosures

